# FLIP use in achalasia: comparing POEM and Heller myotomy outcomes: a systematic review and meta-analysis

**DOI:** 10.1007/s00464-025-11776-4

**Published:** 2025-05-21

**Authors:** Mohammad Alabbas, Hamza Khoudari, Gaurav Ghosh, Omar T. Sims, David Wan

**Affiliations:** 1 Department of Gastroenterology, Hepatology and Nutrition, Cleveland Clinic, 9500 Euclid Avenue, Cleveland, OH 44195 USA; 2 Internal Medicine Department A, University of Debrecen, Debrecen, Hungary; 3 Division of Gastroenterology and Hepatology, New York-Presbyterian Hospital/Weill Cornell Medicine, New York, NY USA; 4 Department of Quantitative Health Sciences, Cleveland Clinic, Cleveland, OH USA; 5 Cleveland Clinic Lerner College of Medicine, Case Western Reserve University School of Medicine, Cleveland, OH USA

**Keywords:** Achalasia, Functional lumen imaging probe, Myotomy, Distensibility index, POEM, Laparoscopic Heller myotomy

## Abstract

**Background:**

Achalasia is a debilitating esophageal motility disorder characterized by absent peristalsis and incomplete relaxation of the lower esophageal sphincter (LES). Myotomy procedures—primarily peroral endoscopic myotomy (POEM) and laparoscopic Heller myotomy (LHM)—aim to reduce LES pressure and alleviate dysphagia. The functional lumen imaging probe (FLIP) measures real-time changes in esophagogastric junction (EGJ) compliance and distensibility during myotomy. We conducted a systematic review and meta-analysis of peer-reviewed literature on the use of preprocedural and intraprocedural FLIP in guiding myotomy adequacy and their impact on clinical outcomes.

**Methods:**

We conducted a systematic review and meta-analysis adhering to PRISMA and Cochrane guidelines. Literature searches (PubMed, Scopus, Cochrane CENTRAL, Web of Science, Google Scholar) were performed through December 2023. We included studies (*N* = 21) assessing FLIP use in achalasia patients undergoing POEM or LHM. Primary outcomes included FLIP measures (distensibility index [DI], cross-sectional area [CSA], diameter [Dmin]) and clinical outcomes (Eckardt score improvement, reflux esophagitis).

**Results:**

A total of 1455 patients were analyzed (mean age 52.3 years; 52.8% male). Both POEM and LHM led to significant increases in DI, CSA, and Dmin at 40 mL FLIP distension (mean differences of 4.69, 100.35, and 4.90, respectively; *p* < 0.001). Eckardt scores significantly decreased after myotomy (MD = − 5.40; 95% CI − 5.91 to − 4.88), with POEM yielding a larger reduction than LHM (*p* = 0.03). Reflux esophagitis occurred in 28% of patients overall—31% following POEM versus 11% following LHM. Intraoperative FLIP was associated with lower esophagitis rates compared to preoperative FLIP use (26% vs. 46%, *p* < 0.05).

**Conclusions:**

FLIP-guided myotomy improves objective EGJ distensibility metrics and aligns with enhanced symptom relief in achalasia. POEM offers greater Eckardt score reductions but carries a higher risk of reflux esophagitis than LHM. Future prospective studies should standardize FLIP protocols, define optimal DI cutoffs, and assess long-term outcomes to further refine achalasia management.

**Supplementary Information:**

The online version contains supplementary material available at 10.1007/s00464-025-11776-4.

Achalasia is a rare yet debilitating esophageal motility disorder marked by absent normal esophageal peristalsis and incomplete relaxation of the lower esophageal sphincter (LES) during swallowing. This leads to the development of dysphagia, regurgitation, and chest pain in patients. Currently, pneumatic dilation, laparoscopic Heller myotomy (LHM), and peroral endoscopic myotomy (POEM) are the most employed treatments. These procedures are designed to alleviate symptoms by reducing the pressure of the lower esophageal sphincter (LES), thereby facilitating esophageal outflow [1, 2]. Assessing the adequacy of myotomy performed during LHM or POEM presents a complex challenge, as postoperative dysphagia can persist when myotomy is inadequate. Conversely, patients may be at risk of developing gastroesophageal reflux disease (GERD) and esophagitis if the myotomy is excessive [3].

The functional lumen imaging probe (FLIP) is a catheter-based endoscopic system designed to assess esophageal motility and the distensibility index (DI) at the esophagogastric junction (EGJ). This measurement provides insights into the compliance of the lower esophageal sphincter (LES) [4]. Patients with achalasia may undergo functional luminal imaging prior to and/or during peroral endoscopic myotomy (POEM) or laparoscopic Heller myotomy (LHM) to obtain real-time assessment of luminal shape and lower esophageal sphincter (LES) function. In contrast to controls, achalasia patients exhibit significantly lower esophageal junction diameter (EGJ DI) and compliance, and functional luminal imaging measurements after myotomy often demonstrate a marked increase in EGJ DI relative to preoperative or early intraoperative assessments [5]. Despite this, data on whether intraoperative FLIP use translates to improved patient-centered outcomes (e.g., reduced dysphagia, chest pain, regurgitation) remains limited. Beyond simply measuring EGJ compliance, FLIP can be applied at different stages of achalasia management. *Intraoperatively*, FLIP measurements of the distensibility index (DI), minimum diameter (Dmin), and cross-sectional area (CSA) can guide the extent of myotomy in real-time. *DI* is derived by dividing the luminal CSA (in mm^2^) by intrabag pressure (in mmHg), reflecting EGJ compliance *Dmin* indicates the narrowest diameter of the EGJ, and *CSA* quantifies luminal opening at a given distension volume [4]. Surgeons can therefore tailor the myotomy length and depth based on these objective parameters, aiming to achieve improved dysphagia outcomes while limiting the risk of postoperative gastroesophageal reflux.

We conducted a systematic review and meta-analysis of peer-reviewed literature on the use of preprocedural and intraprocedural FLIP in guiding myotomy adequacy and their impact on clinical outcomes, specifically symptom improvement (as measured by Eckardt score) and the incidence of reflux esophagitis.

## Methods

### Literature search

We followed the guidelines of the Preferred Reporting Items for Systematic Reviews and Meta-Analysis (PRISMA) statement [6] to conduct a comprehensive and rigorous review of published literature. The methods employed were in strict compliance with the Cochrane Handbook of Systematic Reviews and Meta-Analysis of Interventions (version 5.1.0). We identified potentially relevant articles from inception to December 2023 using the following databases: PubMed, Scopus, Cochrane Library (CENTRAL database), Web of Science, and Google Scholar. We utilized a combination of specific keywords and Medical Subject Headings (MeSH terms) related to “achalasia,” “functional lumen imaging probe” (FLIP), "peroral endoscopic myotomy" (POEM), “laparoscopic Heller myotomy” (LHM), and patient outcomes such as “distensibility index,” “cross-sectional area,” and “Eckardt score” (Supplementary Table 1). The search strategy included terms like “achalasia AND FLIP,” “achalasia AND POEM,” “achalasia AND LHM,” “achalasia AND distensibility index,” among others. Additionally, we manually searched the references of the included studies (backward citation analysis) and relevant reviews to ensure all pertinent literature was identified and considered. This process was conducted in the following manner: (1) screening the reference list of finally included studies following the study selection process, (2) searching relevant articles using PubMed’s “similar articles” function, and (3) searching Google software for relevant articles using the keywords that were employed in the database search.

### Primary outcomes

The primary outcomes of this meta-analysis include FLIP-related measures—namely the Distensibility Index (DI), Cross-Sectional Area (CSA), and minimum diameter (Dmin) at 40 mL—as well as clinical outcomes encompassing symptom improvement (Eckardt score reduction) and the incidence of reflux esophagitis.

### Inclusion and exclusion criteria

We included randomized controlled trials (RCTs), non-randomized controlled trials (NCTs), cohort studies, case–control studies, and case series with a minimum of 20 patients (to avoid inclusion of studies that may have insufficient statistical power), including adult patients with achalasia who underwent myotomy and FLIP either before or during the procedure. We excluded the following studies: (1) case reports, editorials, commentaries, letters to the editor, and conference abstracts; (2) those that did not specifically report FLIP measurements before or during myotomy procedures; (3) those that did not report on myotomy adequacy or postoperative patient outcomes; and (4) those that did not include sufficient data for analysis or where relevant data could not be extracted. Studies were included if they provided at least one FLIP measurement performed either preoperatively or intraoperatively and at least one subsequent postoperative measurement (immediately after myotomy, or at any follow-up interval) to allow for paired comparisons of EGJ distensibility.

### Data extraction

Independent reviewers initially screened titles and abstracts to determine eligibility for inclusion (M.A., H.K.). Subsequently, full-text screening was conducted of studies that met eligibility criteria. Any disagreements were resolved through discussion or by consulting a third reviewer (G.G.). Data from the included studies were extracted and recorded in a standardized data extraction sheet. The extracted data encompassed four main categories: (1) characteristics of the included studies, (2) characteristics of the study population, (3) risk of bias domains, and (4) outcome measures. Data were extracted for a series of comparisons to be conducted as follows: (1) preoperative vs. postoperative outcomes, (2) FLIP vs. control no FLIP or other intraoperative assessment techniques. We extracted adequacy of myotomy assessed intra-operatively using FLIP (e.g., changes in DI and diameter), postoperative patient outcomes (e.g., reduction in dysphagia, regurgitation, chest pain, and recurrence of achalasia), and complication rates (e.g., postoperative dysphagia, reflux esophagitis, length of hospital stay, or need for additional interventions or surgeries).

### Quality assessment

Risk of bias in any randomized controlled trials would have been assessed with the Cochrane RoB 2 tool [7], and non-randomized studies with ROBINS-I [8]; because all eligible studies were observational, we ultimately applied the Newcastle–Ottawa Scale (NOS). The NOS assigns up to nine stars based on three domains: selection (0–4 stars), comparability (0–2 stars), and outcome (0–3 stars). A study is rated high quality (7–9 stars) if it demonstrates a well-defined cohort, thorough control of potential confounding factors, and sufficient outcome measures with adequate follow-up. A study is considered moderate quality (5–6 stars) if it partially meets these criteria—for example, less rigorous confounding control or a more limited follow-up period. Studies earning fewer than 5 stars are classified as low quality. Two independent reviewers (e.g., M.A. and H.K.) applied the NOS, with disagreements resolved by a third reviewer (G.G.).

### Data synthesis and analysis

The data retrieved from the included studies were qualitatively summarized and quantitatively analyzed. For continuous outcomes, the mean command was used to pool the mean difference (MD) and its corresponding 95% confidence interval (CI) across studies [9]. Meanwhile, for dichotomous outcomes with no comparison group (such as clinical success), the metaprop command was used to pool the reported effect size (ES) and its 95% CI from included studies. [10] The selection of the statistical model and method was based on the observed statistical heterogeneity and the nature of the analyzed outcome (i.e., dichotomous or continuous). Statistical heterogeneity was defined as an I2 of > 50% with a *P*-value < 0.05. In case of heterogeneity, the random-effects model was used; otherwise, the fixed-effects model was used [11]. For each outcome, three different subgroup analyses were performed: (1) based on the type of myotomy (POEM or LHM), (2) based on the timing of pre-myotomy FLIP measurement (preoperative or intraoperative), and (3) based on post-myotomy FLIP measurement (i.e., immediately after myotomy completion or after months of surgery). The assessment of publication bias, through funnel plots and Egger’s regression test, was not feasible due to the low number of studies analyzed in each outcome (< 10 studies). Of note, data transformation was carried out in multiple studies. [12–19] to change data from being reported as median (interquartile range or range) into mean (standard deviation) using validated equations [20, 21]. STATA Software (Version 18) was used to run meta-analysis.

## Results

### Database search results

**Fig. 1:**
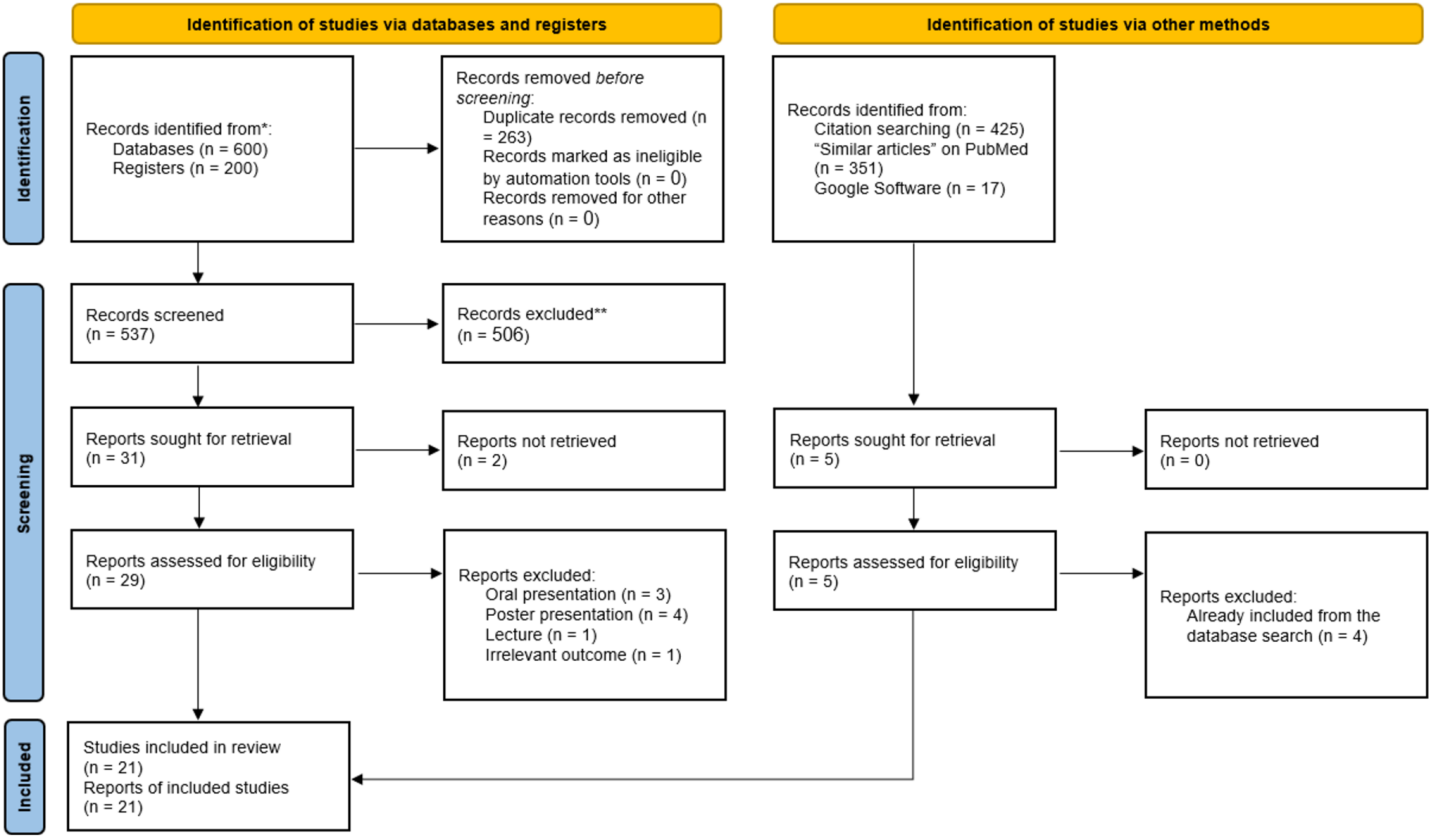
PRISMA Flow Diagram of the study selection process. The diagram details the number of records identified through database searches, duplicate removal, screening of titles/abstracts, reasons for exclusion (e.g., oral presentation, poster presentation, lecture, irrelevant outcomes), and the final number of studies (*N* = 21) included in the meta‐analysis

The initial search yielded 800 titles (Fig. 1). After importing them into EndNote for duplicate identification, 263 records were excluded. The remaining records were then imported into an Excel sheet for screening. The screening of titles and abstracts resulted in 31 potentially eligible articles, out of which two did not have full-text articles available, and thus, they were excluded. Among the 29 remaining articles, nine studies were excluded for the following reasons: oral presentation (*n* = 3), poster presentation (*n* = 4), lecture (*n* = 1), and reporting irrelevant outcomes (*n* = 1), and reporting irrelevant outcomes (*n* =). The manual search process yielded one additional article that was not found through the database search, [22] resulting in a total of 21 studies eligible for data synthesis [9, 12–19, 22–34].

### Baseline characteristics

Of the 21 studies, 4 were prospective and non-experimental, and 17 were retrospective (Table 1). Thirteen studies were conducted in the United States, two in the United Kingdom, five in South Korea, and one in Italy. These studies reported FLIP data of 1455 patients with achalasia undergoing either POEM (*n* = 20) or LHM (*n* = 7). Of these patients, 29.66%, 56.87%, and 13.47% had type I, II, and III achalasia, respectively. The majority of patients were males (52.78%) with a mean age of 52.31 ± 6.70 years. More than one-fourth (27.62%) of patients had prior treatment before POEM or LHM. The follow-up period ranged from 1.5 to 12 months with a mean duration of 11.33 ± 11.50 months. Regarding FLIP data, the first measurement was performed preoperatively in three studies and intraoperatively in 18 studies. Meanwhile, the second FLIP measurement was done immediately after myotomy completion in 16 studies, 6–12 months after myotomy in two studies, and 1 month after myotomy in two studies. Notably, the majority of included studies used the standard 8-cm FLIP probe (EF-325), though a few more recent investigations employed a 16-cm panometry catheter. Despite these variations, the core measurement of EGJ distensibility is comparable across devices.

**Table 1:** Baseline characteristics of included studies discussing the use of FLIP in myotomy among achalasia patients

Author (YOP)	Country	Design	Intervention	Achalasia type (*n*)			Sample (*n*)	Age (years)	Gender		Follow-up (months)	Prior treatment (*n*)
				I	II	III		Mean ± SD	Male	Female		
Attaar (2021)	USA	Retrospective cohort	POEM	6	18	12	43	65 ± 16	23	20	12	–
Campagna (2021)	USA	Retrospective cohort	POEM	27	58	16	71	53	67	47	55	26
Chang (2020)	Korea	Retrospective cohort	POEM	83	91	21	195	43.75 ± 15.53	93	102	9.62	85
Familiari (2014)	Italy	Prospective cohort	POEM	13	10	–	23	51.7	12	11	5	–
Gong (2021)	South Korea	Prospective cohort	POEM	8	9	3	20	43	7	13	11.9	6
Goong (2020)	South Korea	Retrospective cohort	POEM	6	14	3	23	46.7 ± 15.1	11	12	3	5
Holmstrom (2021a)	USA	Retrospective cohort	POEM	6	24	2	35	53 ± 21.17	23	12	12	9
			LHM	6	4	1	11	53 ± 35.79	4	7	12	5
Holmstrom (2021b)	USA	Retrospective cohort	POEM	30	68	27	142	57	77	65	12	30
Holmstrom (2021c)	USA	Retrospective cohort	POEM	15	36	4	61	54	43	18	12	7
Hsing (2022)	South Korea	Retrospective cohort	POEM	14	39	15	68	521 ± 7	33	35	6.83	–
Ilczyszyn (2016)	UK	Retrospective cohort	LHM	8	7	–	15	40	4	11	–	5
Ngamruengphong (2016)	USA	Retrospective cohort	POEM	13	42	5	63	48.3 ± 16.3	32	30	4.06	29
Su (2020a)	USA	Retrospective cohort	POEM	–	–	–	94	–	–	–	–	–
			LHM	–	–	–	9	–	–	–	–	–
Su (2020b)	USA	Prospective cohort	ALL	11	39	21	77	65 ± 16.1	47	30	12	29
			POEM	11	35	20	71	65.7 ± 15.9	42	29	–	28
			LHM	0	4	1	6	56.8 ± 18.1	5	1	–	1
Teitelbaum (2013)	USA	Retrospective cohort	POEM	4	9	1	14	48 ± 15	9	5	–	1
			LHM	4	5	1	11	53 ± 16	6	5	–	6
Teitelbaum (2015)	USA	Retrospective cohort	POEM	11	21	1	36	50 ± 15	25	11	11	7
			LHM	6	10	1	20	53 ± 14	9	11	12	8
Yoo (2019)	South Korea	Retrospective cohort	POEM	21	26	5	52	42.5 ± 15.2	27	25	–	22
Amundson (2023)	USA	Retrospective cohort	POEM	7	17	8	35	61 ± 19	17	18	5	11
DeWitt (2022)	USA	Retrospective cohort	POEM	10	68	9	87	51.3 ± 17	50	37	7.2	–
Knight (2022)	UK	Prospective cohort	POEM	31	83	16	142	50.4	84	58	1.5	74
Teitelbaum (2014)	USA	Retrospective cohort	POEM	5	12	2	19	49 ± 16	13	6	–	4
			LHM	4	7	1	12	54 ± 14	5	7	–	4

*YOP* Year of publication, *UK* United Kingdom, *USA* United States of America, *POEM* Peroral endoscopic myotomy, *LHM* Laparoscopic Heller myotomy, *SD* Standard deviation

This table summarizes the key characteristics of the 21 studies included in the meta-analysis. For each study, the following information is provided: Author (YOP): First author with year of publication; Country: Country of the study; Design: Study design (e.g., retrospective or prospective cohort); Intervention: Type of myotomy procedure (e.g., POEM or LHM); Achalasia Type (n): Number of patients with achalasia subtypes I, II, and III (if reported); Sample (n): Total number of patients; Age (years): Mean age ± standard deviation; Gender: Number of males and females; Follow-up (months): Prior Treatment (n): Number of patients with any prior treatment for achalasia Duration of postoperative follow-up

### Risk of bias assessment

Following the assessment using the Newcastle–Ottawa Scale, 62% (13/21) of the included studies were rated as high quality, whereas 38% (8/21) were rated as moderate quality (Table 2). All studies were deemed methodologically sound and were included in the current meta-analysis.

**Table 2:** Risk of bias assessment of included cohort studies using the New Castle Ottawa Scale for Observational Studies

Author (YOP)	Selection				Comparability		Outcome			Overall rating
	Representativeness of the exposed cohort	Selection of non-exposed cohort	Ascertainment of exposure	Demonstration of the outcome of interest	Control for age, sex, and marital status	Control for other confounding factors	Assessment	Follow-up	Adequacy of follow-up	
Attaar (2021)	★	–	★	★	★	–	★	★	★	High quality
Campagna (2021)	★	–	★	★	–	–	★	★	★	Moderate quality
Chang (2020)	★	–	★	★	–	–	★	★	★	Moderate quality
Familiari (2014)	★	–	★	★	★	–	★	★	★	High quality
Gong (2021)	★	★	★	★	–	–	★	★	★	High quality
Goong (2020)	★	★	★	★	★	–	★	★	★	High quality
Holmstrom (2021a)	★	–	★	★	–	–	★	★	★	Moderate quality
Holmstrom (2021b)	★	★	★	★	–	–	★	★	★	High quality
Holmstrom (2021c)	★	★	★	★	★	★	★	★	★	High quality
Hsing (2022)	★	–	★	★	★	-	★	★	★	High quality
Ilczyszyn (2016)	★	★	★	★	★	★	★	★	★	High quality
Ngamruengphong (2016)	★	–	★	★	–	–	★	★	★	Moderate quality
Su (2020a)	★	★	★	★	–	–	★	★	★	High quality
Su (2020b)	★	–	★	★	–	–	★	★	★	Moderate quality
Teitelbaum (2013)	★	–	★	★	–	–	★	★	★	Moderate quality
Teitelbaum (2015)	★	★	★	★	–	–	★	★	★	High quality
Yoo (2019)	★	★	★	★	–	–	★	★	★	High quality
Amundson (2023)	★	★	★	★	–	–	★	★	★	High quality
DeWitt (2022)	★	–	★	★	–	–	★	★	★	Moderate quality
Knight (2022)	★	★	★	★	–	–	★	★	★	High quality
Teitelbaum (2014)	–	–	★	★	–	–	★	★	★	Moderate quality

*YOP* Year of Publication, High Quality (7–9 stars), Moderate Quality (5–6 stars), Low Quality (1–4 stars)

This table presents the risk of bias assessment for each of the 21 included studies using the NOS for observational studies. The table includes scores (indicated by stars) for the following domains: Selection: Representativeness of the exposed cohort, selection of non‐exposed cohort, and ascertainment of exposure; Outcome: Demonstration of the outcome of interest, assessment of outcome, and adequacy of follow-up; Comparability: Control for age, sex (and marital status, if applicable) as well as for additional potential confounders. An overall rating is provided for each study based on the total NOS score, with studies classified as “High Quality” (7–9 stars) or “Moderate Quality” (5–6 stars)

### FLIP-related outcomes

#### Distensibility at 40 mL

**Fig. 2:**
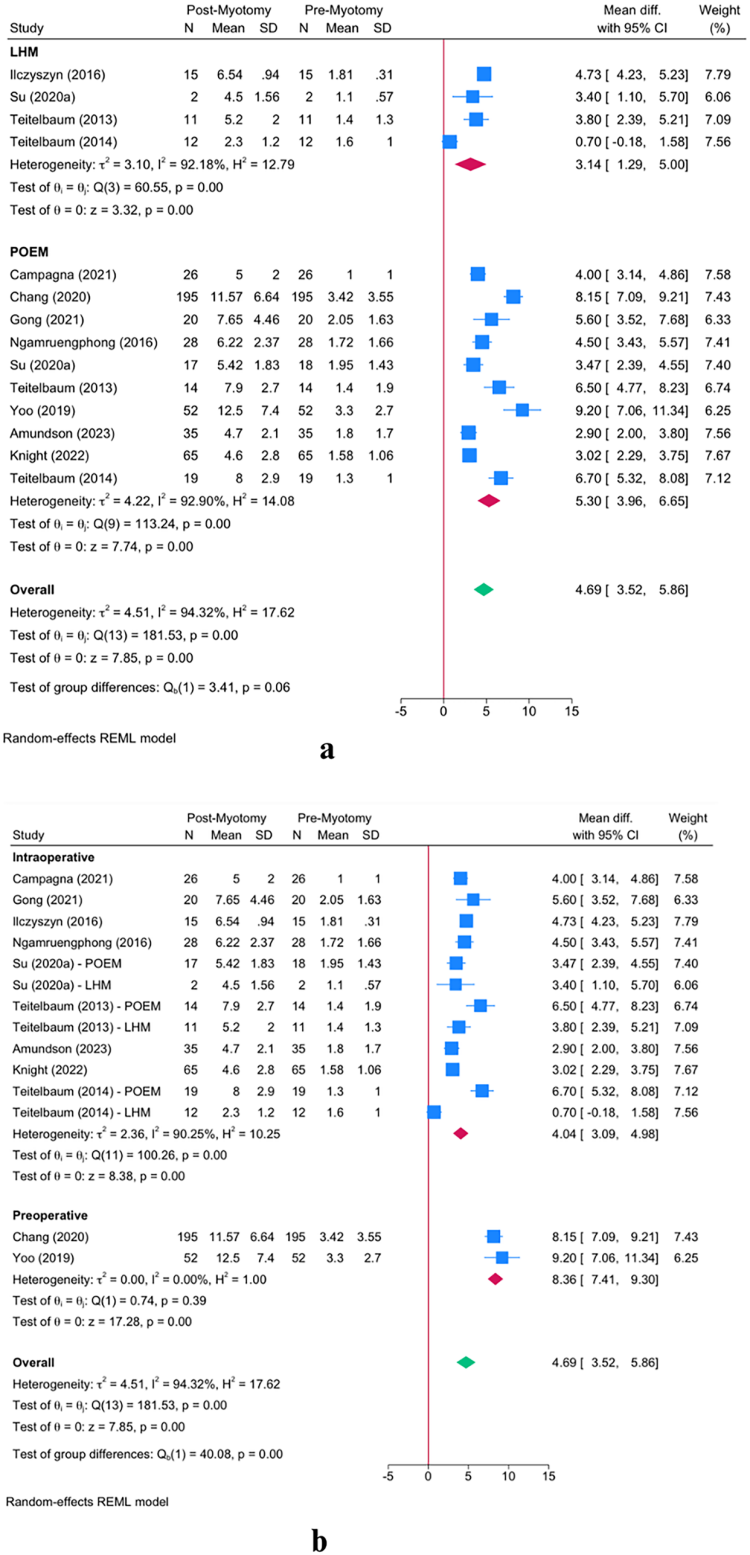

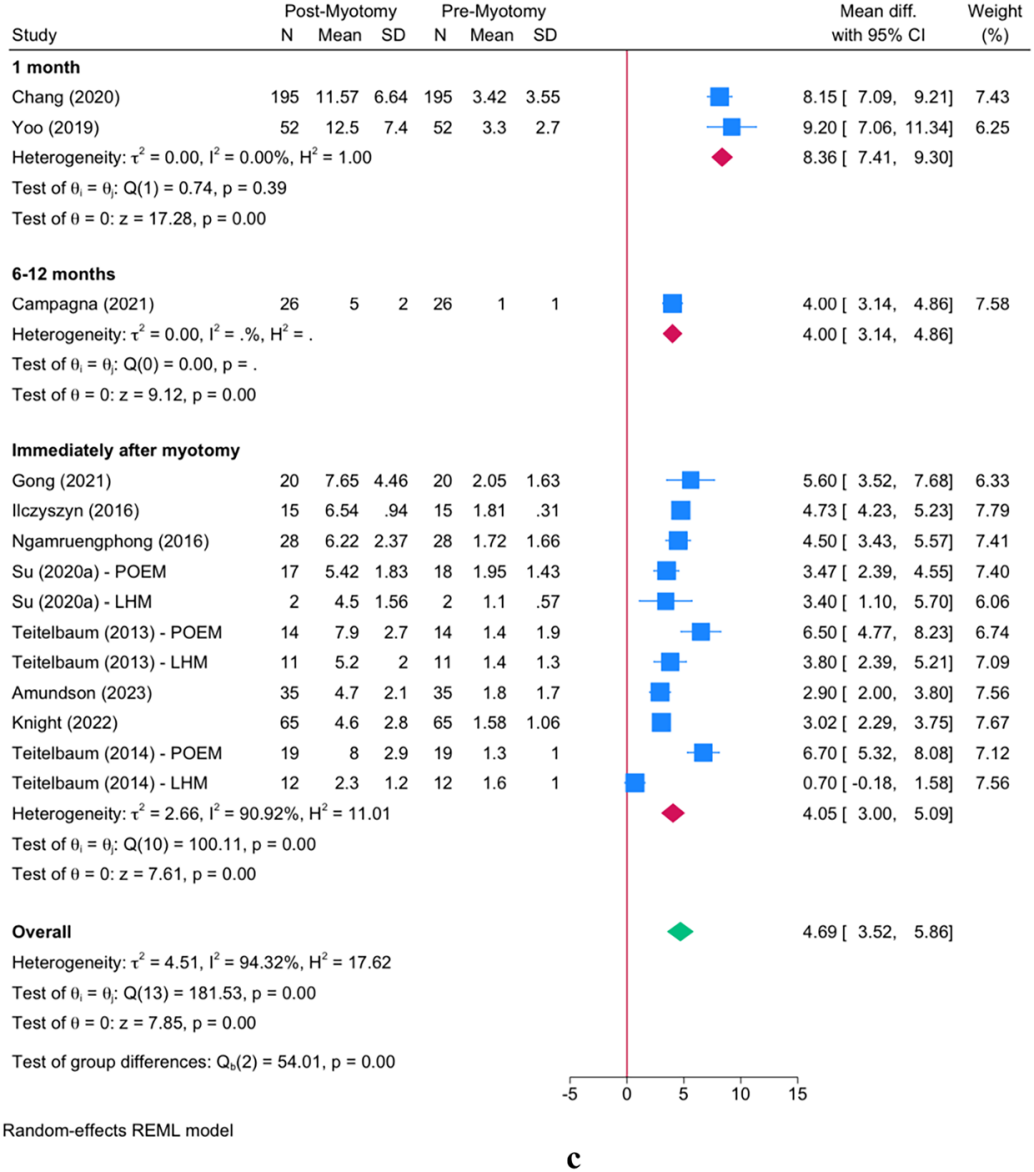
FLIP measurements at a 40 mL distension volume. Panel **a** shows the pre‐myotomy FLIP values, panel **b** presents intraoperative values, and panel **c** depicts post‐myotomy measurements. Error bars represent 95% confidence intervals

FLIP measurement at 40 mL distention showed a significant increase in DI following myotomy among achalasia patients [MD = 4.69; 95% CI 3.52–5.86] (Fig. 2a). This increase was significant in both operations—LHM [MD = 3.14; 95% CI 1.29–5.00] and POEM [mean difference (MD) = 5.30; 95% CI 3.96–6.65]. Although POEM resulted in a greater increase in DI compared to LHM, the difference was not statistically significant (*P* = 0.06).

Next, we observed a significant effect modification on DI at 40 mL based on the timing of FLIP usage (*P* = 0.001). When FLIP was applied intraoperatively [MD = 4.04; 95% CI 3.09–4.98], DI increased less than when FLIP was used preoperatively [MD = 8.36; 95% CI 7.41–9.30] (Fig. 2b).

Similarly, the timing of post-myotomy FLIP measurement demonstrated a significant impact on DI at 40 mL (*P* = 0.001). The subgroup meta-analysis indicated that performing FLIP immediately after myotomy completion resulted in a smaller increase compared to measurements taken one month post-procedure [MD = 4.05; 95% CI 3.00–5.09 vs. MD = 8.36; 95% CI 7.41–9.30, respectively] (Fig. 2c).

#### Cross-sectional area (CSA) at 40 mL

**Fig. 3:**
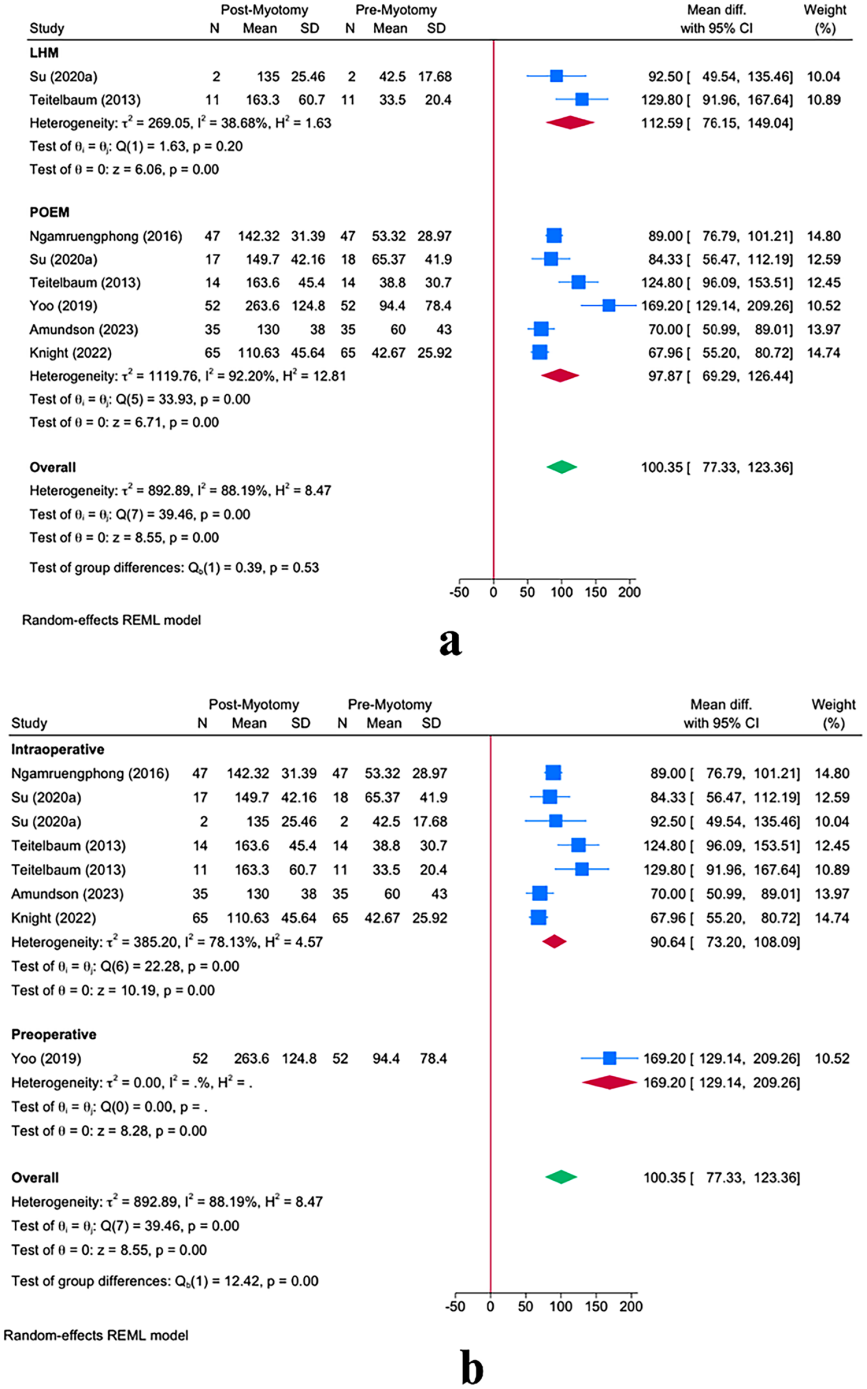

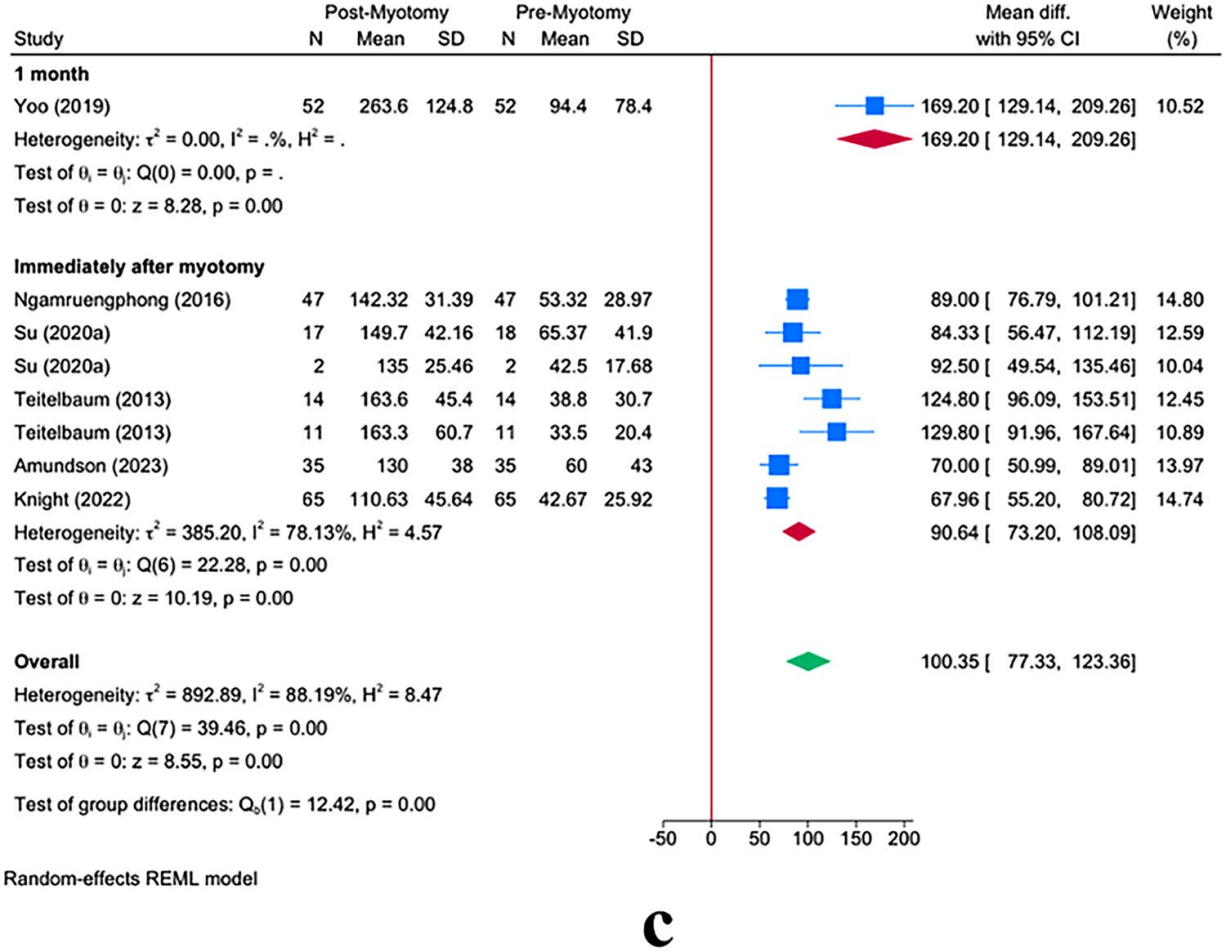
Changes in cross-sectional area (CSA) and Eckardt score: Panel **a** illustrates the overall increase in CSA following myotomy, comparing LHM and POEM groups. Panel **b** focuses on intraoperative versus preoperative FLIP usage and its effect on CSA. Panel **c** compares CSA measurements taken immediately after myotomy versus at 1 month post-myotomy. Error bars represent 95% confidence intervals

Following myotomy, FLIP measurement revealed a significant increase in CSA at 40 mL [MD = 100.35; 95% CI 77.33: 123.36] (Fig. 3a). This increase was significant in both POEM and LHM operations with no significant difference between these procedures (*P* = 0.53).

The timing in which FLIP was used revealed a significant effect modification on CSA at 40 mL (*P* = 0.001). The intraoperative use of FLIP [MD = 90.64; 95% CI 108.09:73.20] was associated with a lesser increase in CSA as compared to preoperative FLIP use [MD = 169.20; 95% CI 209.26: 129.14] (Fig. 3b).

The timing of post-myotomy FLIP measurement also revealed a significant effect modification on CSA at 40 mL *P* = 0.001. The subgroup meta-analysis showed that the immediate use of FLIP after myotomy completion results in a lesser increase as compared to its use at 1 month after myotomy [MD = 90.64; 95% CI 108.09:73.20 vs. MD = 169.20; 95% CI 209.26: 129.41], respectively (Fig. 3c).

#### Diameter min (Dmin) at 40 mL

The FLIP measurement revealed a significant increase in Dmin at 40 mL following myotomy in achalasia patients [MD = 4.90; 95% CI 4.02:5.87] (Supplementary Fig. 9). This increase was consistent and quite similar across POEM and LHM surgeries (*P* = 0.50). The effect modification of time of FLIP measurement could not be assessed as all of the analyzed studies reported the use of intraoperative FLIP alone with no data on preoperative FLIP usage.

### Clinical outcomes

#### Eckardt score

**Fig. 4:**
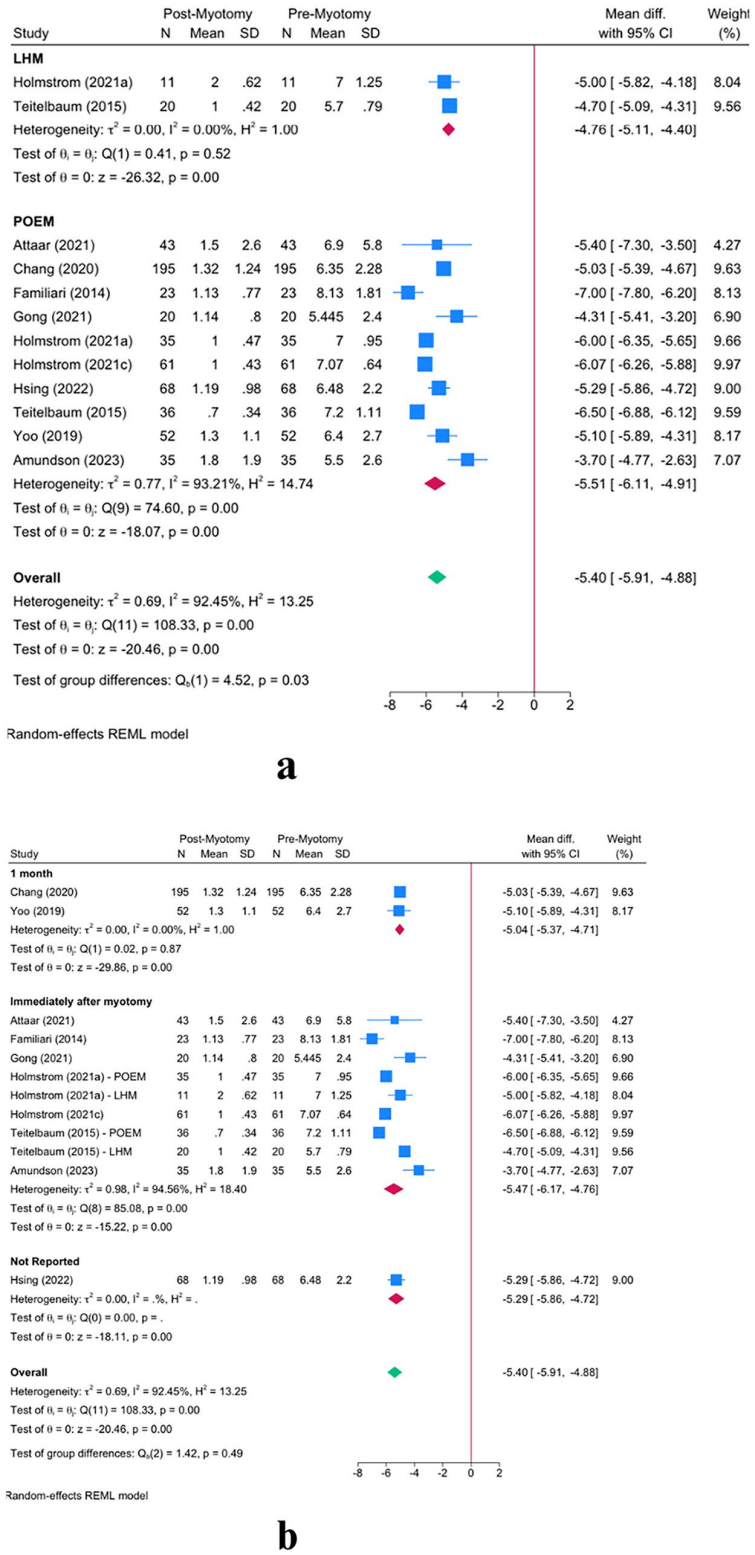

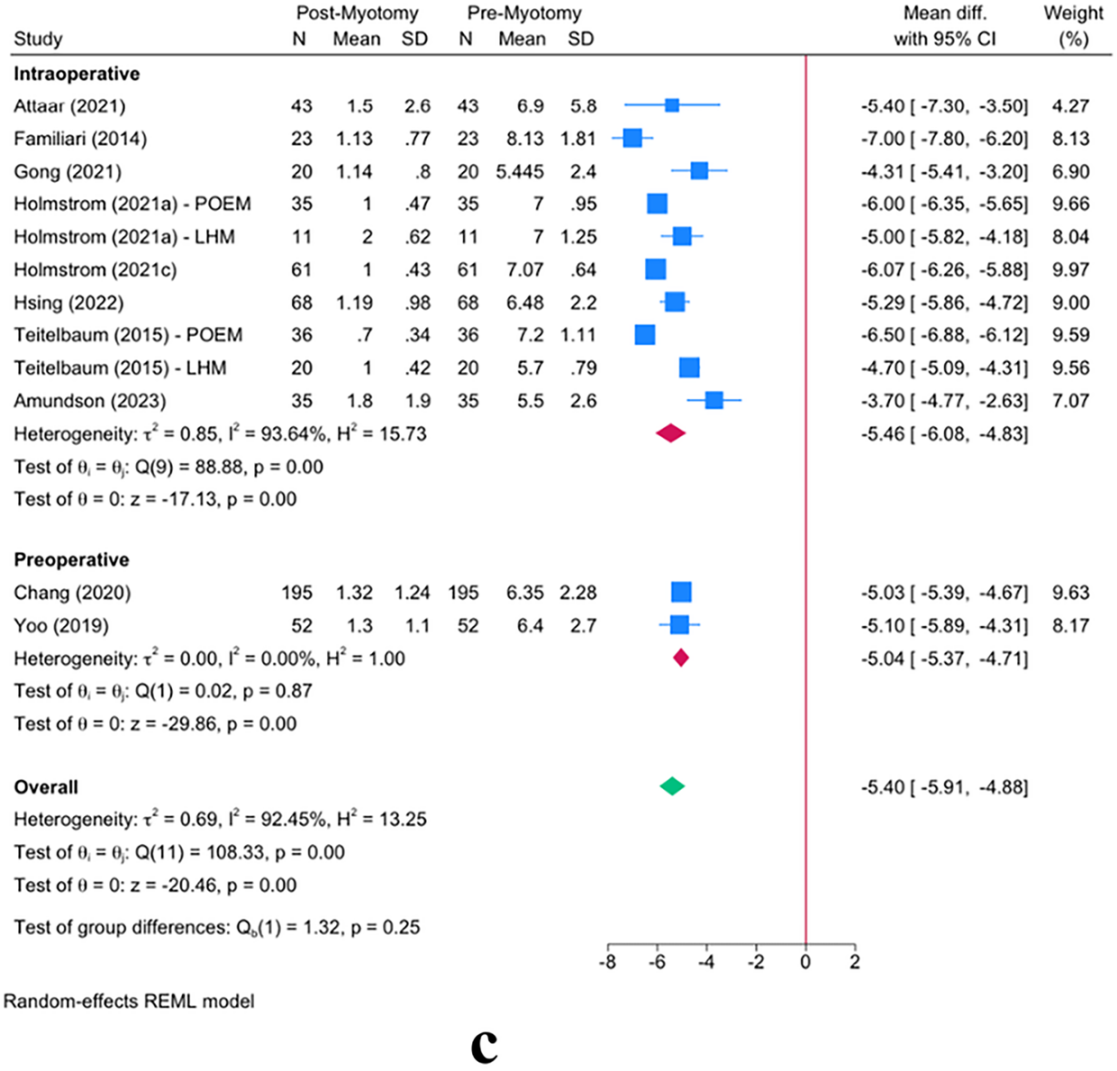
Effect of FLIP timing on distensibility outcomes: Panel **a** shows the overall reduction in Eckardt scores from pre- to post-myotomy, stratified by LHM and POEM. Panel **b** compares immediate postoperative Eckardt-score measurements with those at 1 month or 6–12 months post-myotomy. Panel **c** contrasts Eckardt-score outcomes based on whether FLIP was applied preoperatively or intraoperatively. Error bars denote the 95% confidence intervals

The meta-analysis revealed a significant reduction in Eckardt score following myotomy in achalasia patients [MD = − 5.40; 95% CI − 5.91: − 4.88] (Fig. 4a). This reduction was maintained in both POEM [MD = − 5.51; 95% CI − 6.11: − 4.91] and LHM [MD = − 4.76; 95% CI − 5.11: − 4.40] groups. However, POEM resulted in a significantly greater reduction in Eckardt score *(P* = *0.03).* The timing of FLIP usage (intraoperative vs. preoperative) did not reveal any significant effect modification on the Eckardt score (*P* = 0.25, Fig. 4c). Similarly, the timing of post-myotomy FLIP measurement did not play any effect-modifying role on the Eckardt score (*P* = 0.49, Fig. 4b).

#### Incidence of esophagitis

**Fig. 5:**
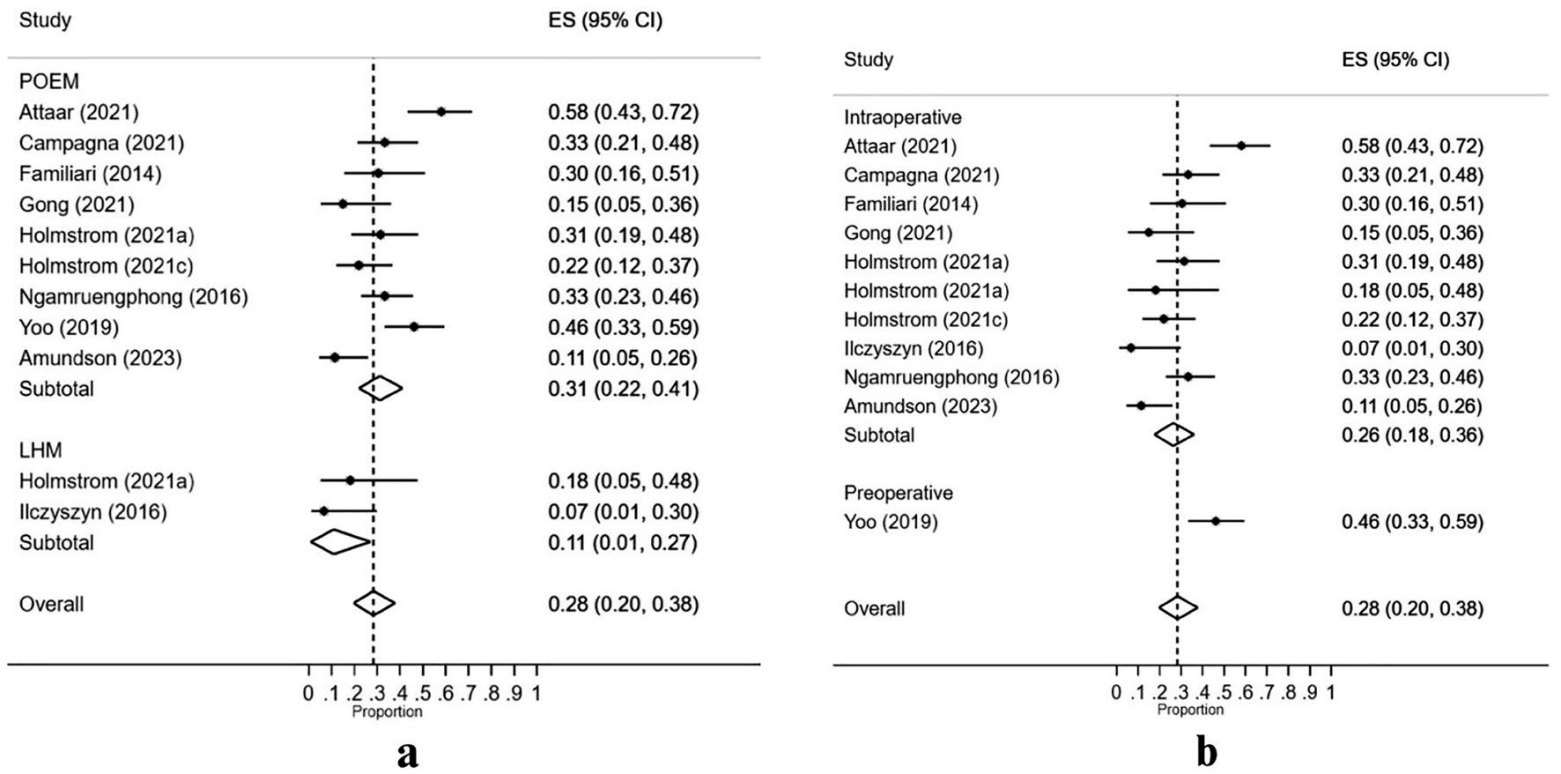
Incidence of postoperative reflux esophagitis: Panel **a** compares the incidence of reflux esophagitis between POEM and LHM groups. Panel **b** compares the incidence of reflux esophagitis according to the timing of FLIP usage (intraoperative versus preoperative). Error bars indicate the 95% confidence intervals

The overall rate of reflux esophagitis was 28% [95% CI 20–38%]. However, this rate was higher in patients undergoing POEM [ES = 31%; 95% CI 22–41%] by almost threefold as compared to those undergoing LHM [ES = 11%; 1–27%] (Fig. 5a).

The timing of FLIP use exhibited an effect modification on the incidence of esophagitis following myotomy in achalasia patients with intraoperative FLIP use [ES = 26%; 95% CI 18–36%] being associated with lower esophagitis rates as compared to its preoperative use [ES = 46%; 95% C 33–59%] (Fig. 5b).

Among our included studies, only Attaar et al [23] explicitly reported the LA classification of postoperative reflux esophagitis, finding 25 out of 43 patients (58.1%) with esophagitis after POEM, categorized as LA Grade A (16%), B (20%), C (44%), and D (8%). Additionally, Campagna et al [5] defined “objective GERD” as LA Grade B or worse, reporting an overall rate of 33% at long-term follow-up. The remaining studies did not specify LA grades, preventing a pooled analysis of reflux severity according to the Los Angeles classification.

## Discussion

This systematic review and meta-analysis demonstrate that the intraoperative use of the functional lumen imaging probe (FLIP) significantly enhances the prediction of physiological and clinical outcomes in achalasia patients undergoing laparoscopic Heller myotomy (LHM) or peroral endoscopic myotomy (POEM). Specifically, our results show that the effect of myotomy on FLIP findings does not differ according to surgical approach; whether patients underwent POEM or LHM, FLIP measurements improved consistently. Moreover, we observed a significant increase in the distensibility index (DI), cross-sectional area (CSA), minimum diameter (Dmin), and esophagogastric junction (EGJ) diameter at various distension volumes—findings that mirror previous reports of increased EGJ distensibility following POEM in achalasia [35–37]. This rise in DI correlates with favorable clinical response, as higher DI values have been noted in patients showing good outcomes compared with those who remain untreated or respond poorly [34]. Unlike the Eckardt score—which relies on subjective symptom reporting—FLIP provides real-time, objective measurements of EGJ function and luminal distensibility, yielding a more comprehensive assessment than high-resolution manometry (HRM) or timed barium esophagography (TBE) [14, 38, 39]. FLIP is also advantageous in patients with low integrated relaxation pressure (IRP), where HRM may be less effective [40]. Notably, FLIP can guide the extent of myotomy during surgery, leading to significant symptomatic relief.14 Supporting this, Holmstrom et al. [16] reported higher clinical success rates (93% vs. 81%, *p* < 0.05) in patients for whom FLIP was used intraoperatively. Additionally, changes in CSA and DI measured by FLIP have emerged as strong predictors of postoperative outcomes in achalasia [38].

However, in terms of clinical outcomes, our results indicate a significant effect-modifying role of the type of surgery on achalasia patients’ Eckardt score and the rate of reflux esophagitis following myotomy. This effect could be explained by multiple factors. First, POEM is associated with a lower degree of pain compared to LHM [41]. According to our findings, both LHM and POEM reduced the Eckardt score, indicating an improvement in achalasia symptoms. Although both POEM and LHM significantly improved Eckardt scores, our analysis shows that POEM produced a larger reduction, suggesting that it may offer greater symptom relief than LHM. Several studies have demonstrated that intraoperative FLIP not only measures EGJ distensibility but also actively guides the surgical approach. For instance, Holmstrom et al [16] reported extending or deepening the myotomy if the post-cut DI remained below a certain threshold, which led to a higher rate of clinical success (93% vs. 81%, *p* < 0.05). Likewise, Ilczyszyn et al [18] found that FLIP measurements allowed for shorter laparoscopic myotomies without jeopardizing symptom relief, thus potentially reducing reflux. These findings underscore how DI, CSA, and Dmin can inform real-time decisions, helping surgeons balance adequate LES disruption against the risk of postoperative GERD. Although we noted a greater Eckardt score reduction with POEM overall, most included studies did not report subtype-stratified improvements. Holmstrom et al. [15] observed that type III patients often exhibit lower postoperative DI levels but can still achieve symptom relief. Consequently, it remains unclear whether FLIP usage confers equal benefit across all subtypes or if Type III patients require more extended myotomy.

However, our findings suggest that a higher risk of esophagitis and reflux diseases are disadvantages of POEM. This could be interpreted as a trade-off between symptom relief efficacy and a higher risk of certain complications. The decision between LHM and POEM may then be influenced by a number of factors, including patients’ specific characteristics and preferences, as well as the expertise of the medical team. In terms of other clinical outcomes, our study highlights a favorable clinical success following myotomy in achalasia of 93% with a low complication rate of 10%; however, the rate of post-myotomy GERD was quite high, accounting for 52% of analyzed patients. It should be noted that these findings are only related to post-POEM achalasia patients, given the scarcity of data regarding LHM. In the same context, our findings show that POEM is associated with a reduced risk of presenting with reflux symptoms as compared to the pre-myotomy period.

The timing of FLIP application varies widely, with some studies using it preoperatively [25, 26, 34] and others intraoperatively [9, 12–19, 22–24, 27–33]. Whether FLIP timing influences patient outcomes remains unclear, as prior systematic reviews only examined intraoperative FLIP [14, 42]. To address this, we conducted a subgroup meta-analysis showing that preoperative FLIP was linked to greater increases in DI and CSA at 40 mL, whereas intraoperative FLIP was associated with a lower incidence of reflux esophagitis. Although this timing effect did not extend to clinical success or Eckardt scores, our findings suggest that using FLIP during myotomy may help reduce excessive dissection and subsequent GERD. Interestingly, intraoperative FLIP was tied to higher subjective reflux reports—aligning with GERD studies indicating that reflux symptoms can persist (or remain absent) irrespective of endoscopic findings. Consequently, esophagitis often serves as a more objective marker for GERD, particularly in patients with chronic esophageal symptoms [43].

Intraoperative FLIP use resulted in a greater reduction in post-myotomy IRP than preoperative FLIP application, and similar timing effects were observed in postoperative FLIP measurements (immediate vs. 1 month), suggesting a potential role in creating a durable myotomy—an underexplored concept. Further research is needed to confirm whether these findings persist, as most studies emphasize short-term outcomes. Importantly, assessing EGJ physiology post-myotomy is critical for estimating long-term results and deciding on reintervention [14]. Only one study in our review addressed reintervention rates: Holmstrom et al. [17] reported that 34 of 61 patients required further intervention after POEM. Data on predictive factors remain limited; hence, future studies should clarify reintervention rates for POEM or LHM and identify predictors such as achalasia subtype, Eckardt score, and FLIP metrics. Establishing an ideal intraoperative FLIP threshold to achieve symptom relief without reflux would be optimal, yet current evidence is inconclusive. For instance, Teitelbaum et al. [31–33] proposed an intraoperative DI range of 4.5–8.5 mm^2^/mmHg for LHM and POEM, associated with an Eckardt score < 1 and a GERD score < 7,33 while in a 54-patient cohort, a 2.8 mm^2^/mmHg cutoff for EGJ DI predicted early success (Eckardt < 3) with an AUC of 0.864.44 [44]. Small sample sizes and short follow-ups limit these findings, underscoring the need for larger, standardized studies with extended observation. In our analysis, patients with a good clinical response had higher EGJ DI at 40 mL (FLIP 325 balloon), whereas CSA at 40 mL did not correlate with improvement—perhaps reflecting differing definitions of “good” outcome (e.g., Eckardt < 1 in Familiari et al. [12] vs. < 3 in Ngamruengphong et al. [28]). In terms of intervention failure, only a few studies in our analysis systematically reported reintervention rates. Holmstrom et al. [17] noted that 34 of 61 patients eventually required additional therapy post-POEM, but a subset analysis suggested that low intraoperative DI predicted the need for further interventions. Other investigators have likewise observed that inadequate DI increase during surgery may signal incomplete myotomy and higher failure risk. [16, 18] This highlights the potential for intraoperative FLIP to reduce reinterventions by guiding a more complete initial myotomy. While a few studies measured FLIP both preoperatively and intraoperatively in the same patients, direct comparisons remain limited. Sedation or anesthesia may alter baseline EGJ compliance; therefore, intraoperative FLIP values might differ from awake preoperative measurements. Further research is needed to establish whether these measurements are fully comparable within the same individual.

Our meta-analysis had several limitations. First, all of the available studies in the literature were observational in design, often lacking a comparison group (FLIP vs. no FLIP). Second, none of the analyzed studies made direct comparisons based on the timing of the FLIP application; thus, our results are entirely based on between-study comparisons, which could not account for all clinical and statistical heterogeneity. Importantly, studies reported different FLIP procedure protocols with different definitions for clinical success. This needs to be standardized so that reported findings in the literature can be reproduced. Third, we could not determine the effect modification of various other factors on distensibility among studied patients, such as achalasia subtype, age, gender, or the presence of prior treatment. Fourth, the majority of LHM studies did not report data related to clinical response/outcomes such as esophagitis development, making it difficult to reach conclusions regarding the effect of modification of surgery on patients’ outcomes. Future investigations should consistently incorporate LA grading to permit a more detailed examination of esophagitis severity following myotomy.

Despite these limitations, our meta-analysis had several notable strengths. The cumulative sample size of 1,455 achalasia patients provided robust statistical power and increased generalizability. Although the majority of included studies were retrospective, the inclusion of prospective studies enhances the quality of evidence by mitigating potential biases inherent in retrospective designs. All studies utilized standardized assessment tools—namely, FLIP measurements (DI, CSA, Dmin) and the Eckardt score—ensuring consistent evaluation and facilitating comparative analyses. Diverse geographic representation, including studies from the United States, United Kingdom, South Korea, and Italy, enhances the applicability of the findings across different populations. Evaluating both POEM and LHM procedures allowed for comparisons between surgical techniques regarding FLIP measurements and patient outcomes. Significant improvements in FLIP measurements and reductions in Eckardt scores post-myotomy suggest that FLIP may play a valuable role in assessing myotomy adequacy and predicting patient outcomes.

## Conclusions

Our results support the use of FLIP during myotomy to improve outcomes in achalasia patients undergoing POEM or LHM. The meta-analysis highlights the importance of FLIP in predicting treatment outcomes following myotomy. However, the precise FLIP threshold for defining clinical success remains undetermined. While our analysis provides greater confidence compared to previous studies, there is similar statistical heterogeneity. Additionally, the timing of FLIP application can significantly influence post-myotomy FLIP measurements and clinical outcomes. The type of surgery may also modify patients’ post-myotomy outcomes. Therefore, further research stratifying patients by characteristics such as achalasia subtype, age, and prior treatment is necessary.

## Supplementary Information

Below is the link to the electronic supplementary material.

Supplementary file1 (DOCX 9 KB)

Supplementary file2 (DOCX 8 KB)

Supplementary file3 (DOCX 9 KB)

## Data Availability

Data, Analytic methods, and study materials can be made available upon request to the corresponding author.

## Abbreviations

DI: Distensibility index
EGJ: Esophagogastric junction
FLIP: Functional lumen imaging probe
GERG: Gastroesophageal reflux disease
LHM: Laparoscopic Heller myotomy
Dmin: Minimum diameter
POEM: Peroral endoscopic myotomy
RCTs: Randomized controlled trials
