# Correction: FLIP use in achalasia: comparing POEM and Heller myotomy outcomes: a systematic review and meta-analysis

**DOI:** 10.1007/s00464-025-11864-5

**Published:** 2025-06-03

**Authors:** Mohammad Alabbas, Hamza Khoudari, Gaurav Ghosh, Omar T. Sims, David Wan

**Affiliations:** 1 Department of Gastroenterology, Hepatology and Nutrition, Cleveland Clinic, 9500 Euclid Avenue, Cleveland, OH 44195 USA; 2 Internal Medicine Department A, University of Debrecen, Debrecen, Hungary; 3 Division of Gastroenterology and Hepatology, New York-Presbyterian Hospital/Weill Cornell Medicine, New York, NY USA; 4 Department of Quantitative Health Sciences, Cleveland Clinic, Cleveland, OH USA; 5 Cleveland Clinic Lerner College of Medicine, Case Western Reserve University School of Medicine, Cleveland, OH USA

**Correction to: Surgical Endoscopy** 10.1007/s00464-025-11776-4

The original online version of this article was revised to correct the attributed affiliation for coauthor David Wan. The correct affiliation is Division of Gastroenterology and Hepatology, New York-Presbyterian Hospital/Weill Cornell Medicine, New York, NY, USA.

The original article has been corrected.

