# Supplementary material for: FLIP use in achalasia: comparing POEM and Heller myotomy outcomes: a systematic review and meta-analysis
Source: Surg Endosc. 2025 May 21;39(7):4060–75. doi: 10.1007/s00464-025-11776-4 (PMC12222239; doi:10.1007/s00464-025-11776-4)
Supplement: Supplementary file 1 — Supplementary file1 (DOCX 9 KB) [file 464_2025_11776_MOESM1_ESM.docx]

**Supplementary Table 1.** The detailed search query implemented in the database search

| Database | No. | Search Query | Results |
| --- | --- | --- | --- |
| PubMed | | | |
|  | #1 | achalasia OR cardiospasm | 9759 |
|  | #2 | myotomy OR POEM OR LHM | 10998 |
|  | #3 | EndoFLIP OR "functional lumen imaging probe" OR "functional luminal imaging probe" OR FLIP | 12172 |
|  | #4 | "patient outcomes" OR outcome* OR dysphagia OR regurgitation OR "chest pain" OR recurrence OR "side effect*" OR "adverse effect*" OR "adverse event*" OR complicat* OR "hospital stay" OR intraoperative OR intra-operative OR perioperative OR peri-operative OR preoperative OR pre-operative OR postoperative OR post-operative | 8331008 |
|  | #5 | #1 AND #2 AND #3 AND #4 | 87 |
| Scopus | | | |
|  | #1 | ALL (achalasia) OR ALL (cardiospasm) | 21395 |
|  | #2 | ALL (myotomy) OR ALL (POEM) OR ALL (LHM) | 161541 |
|  | #3 | ALL (EndoFLIP) OR ALL ("functional lumen imaging probe") OR ALL ("functional luminal imaging probe") OR ALL (FLIP) | 200454 |
|  | #4 | ALL ("chest pain") OR ALL (recurrence) OR ALL ("side effect") OR ALL ("adverse effect") OR ALL ("adverse event") OR ALL (complicated) OR ALL (complication) OR ALL ("hospital stay") OR ALL (intraoperative) OR ALL (intra-operative) OR ALL (perioperative) OR ALL (peri-operative) OR ALL (preoperative) OR ALL (pre-operative) OR ALL (postoperative) OR ALL (post-operative) | 8049176 |
|  | #5 | #1 AND #2 AND #3 AND #4 | 417 |
| Web of Science | | | |
|  | #1 | ALL=achalasia OR ALL=cardiospasm | 9013 |
|  | #2 | ALL=myotomy OR ALL=POEM OR ALL=LHM | 71238 |
|  | #3 | ALL=EndoFLIP OR ALL="functional lumen imaging probe" OR ALL="functional luminal imaging probe" OR ALL=FLIP | 67361 |
|  | #4 | ALL="patient outcomes" OR ALL=outcome* OR ALL=dysphagia OR ALL=regurgitation OR ALL="chest pain" OR ALL=recurrence OR ALL="side effect*" OR ALL="adverse effect*" OR ALL="adverse event*" OR ALL=complicat* OR ALL="hospital stay" OR ALL=intraoperative OR ALL=intra-operative OR ALL=perioperative OR ALL=peri-operative OR ALL=preoperative OR ALL=pre-operative OR ALL=postoperative OR ALL=post-operative | 5544809 |
|  | #5 | #1 AND #2 AND #3 AND #4 | 92 |
| CENTRAL (Cochrane) | | | |
|  | #1 | achalasia OR cardiospasm | 475 |
|  | #2 | myotomy OR POEM OR LHM | 753 |
|  | #3 | EndoFLIP OR "functional lumen imaging probe" OR "functional luminal imaging probe" OR FLIP | 755 |
|  | #4 | "patient outcomes" OR outcome OR dysphagia OR regurgitation OR "chest pain" OR recurrence OR "side effect" OR "adverse effect" OR "adverse event" OR complicated OR complication OR "hospital stay" OR intraoperative OR intra-operative OR perioperative OR peri-operative OR preoperative OR pre-operative OR postoperative OR post-operative | 891379 |
|  | #5 | #1 AND #2 AND #3 AND #4 | 4 |
| Google Scholar | | | |
|  | With all of the words | EndoFLIP myotomy achalasia |  |
|  | With the exact phrase |  |  |
|  | With at least one of the words | "patient outcomes" outcome dysphagia regurgitation "chest pain" recurrence "side effect" "adverse effect" "adverse event" complicated complication "hospital stay" intraoperative intra-operative perioperative peri-operative preoperative pre-operative postoperative post-operative | |
|  | Total | As per recent recommendations, only the first 200 records were screened | 200 |
