# Supplementary material for: FLIP use in achalasia: comparing POEM and Heller myotomy outcomes: a systematic review and meta-analysis
Source: Surg Endosc. 2025 May 21;39(7):4060–75. doi: 10.1007/s00464-025-11776-4 (PMC12222239; doi:10.1007/s00464-025-11776-4)
Supplement: Supplementary file 2 — Supplementary file2 (DOCX 8 KB) [file 464_2025_11776_MOESM2_ESM.docx]

**Supplementary Table 2.** The statistical test and method used in each of the analyzed outcomes

| Outcome | Statistical | |
| --- | --- | --- |
|  | **Model** | **Method** |
| EndoFLIP | | |
| DI 30 mL | Random-effects | REML |
| DI 40 mL | Random-effects | REML |
| DI 50 mL | Fixed-effects | IV |
| CSA (30mL) | Random-effects | REML |
| CSA (40mL) | Random-effects | REML |
| Intrabag pressure (30mL) | Random-effects | REML |
| Intrabag pressure (40mL) | Fixed-effects | IV |
| EGJ diameter (30mL) | Random-effects | REML |
| Dmin (30mL) | Fixed-effects | IV |
| Dmin (40mL) | Fixed-effects | IV |
| Clinical outcomes | | |
| Clinical success (Eckardt score) | Random-effects | REML |
| Complications/Adverse events | Random-effects | REML |
| incidence of GERD | Random-effects | REML |
| Reflux esophagitis | Random-effects | REML |
| Operative time | Random-effects | REML |
| Reflux symptoms | Random-effects | REML |
| HRM data | | |
| LES pressure | Fixed-effects | IV |
| IRP | Fixed-effects | IV |

DI: Distensibility Index; CSA: Cross-Sectional Area; Dmin: Minimum Distensibility; EGJ: Esophagogastric Junction; GERD: Gastroesophageal Reflux Disease; LES: Lower Esophageal Sphincter; IRP: Integrated Relaxation Pressure; HRM: High-Resolution Manometry; IV: Inverse Variance; REML: Restricted Maximum-Likelihood
