# Supplementary material for: FLIP use in achalasia: comparing POEM and Heller myotomy outcomes: a systematic review and meta-analysis
Source: Surg Endosc. 2025 May 21;39(7):4060–75. doi: 10.1007/s00464-025-11776-4 (PMC12222239; doi:10.1007/s00464-025-11776-4)
Supplement: Supplementary file 3 — Supplementary file3 (DOCX 9 KB) [file 464_2025_11776_MOESM3_ESM.docx]

**Supplementary Table 3.** EndoFLIP characteristics including measurement timepoints and inflation protocols reported in the literature

| Author (YOP) | EndoFLIP Characteristics | | | | | |
| --- | --- | --- | --- | --- | --- | --- |
|  | **Procedure** | **Site of inflation** | **Distension** | | **Measurement Timepoints** | |
|  |  |  | **Time** | **Volume** | **First** | **Second** |
| Attaar (2021) | Transoral, general anesthesia; under endoscopic control | EJG and Body | NR | NR | Intraoperative | Immediately after myotomy |
| Campagna (2021) | Preoperative: transoral conscious sedation; under endoscopic control/intraoperative: transoral, general anesthesia, under endoscopic control | EJG and Body | 30-60s | 30, 40, 50, 60, and 70 mL | Intraoperative | 6-12 months |
| Chang (2020) | Transoral, general anesthesia; under endoscopic control | EGJ | 30s | 40mL | Preoperative | 1 month |
| Familiari (2014) | Transoral, general anesthesia; under endoscopic control | EGJ | 30s | 30mL | Intraoperative | Immediately after myotomy |
| Gong (2021) | Transoral, general anesthesia; under endoscopic control | EGJ | 30s | 40-50mL | Intraoperative | Immediately after myotomy |
| Goong (2020) | Transoral, general anesthesia; under endoscopic control | EGJ | 30s | 50mL | Intraoperative | Immediately after myotomy |
| Holmstrom (2021a) | Transoral, general anesthesia; under endoscopic control | EGJ | 40s | 40,60mL | Intraoperative | Immediately after myotomy |
| Holmstrom (2021b) | Transoral, general anesthesia; under endoscopic control | EGJ | 40s | 40,60mL | Intraoperative | Immediately after myotomy |
| Holmstrom (2021c) | Transoral, general anesthesia; under endoscopic control | EGJ | 40s | 40,60mL | Intraoperative | Immediately after myotomy |
| Hsing (2022) | Transoral, general anesthesia; under endoscopic control | EGJ | NR | NR | Intraoperative | - |
| Ilczyszyn (2016) | Transoral, general anesthesia; under endoscopic control | EGJ | 30s | 30-40mL | Intraoperative | Immediately after myotomy |
| Ngamruengphong (2016) | Transoral, general anesthesia; under endoscopic control | EGJ | 30s | 30-40mL | Intraoperative | Immediately after myotomy |
| Su (2020a) | Transoral, general anesthesia; under endoscopic control | EGJ | 30s | 30-40 mL | Intraoperative | Immediately after myotomy |
| Su (2020b) | Transoral, general anesthesia; under endoscopic control | EGJ | 30s | 30-40 mL | Intraoperative | Immediately after myotomy |
| Teitelbaum (2013) | Transoral, general anesthesia; under endoscopic control | EGJ | NR | 30-40-50mL | Intraoperative | Immediately after myotomy |
| Teitelbaum (2015) | Transoral, general anesthesia; under endoscopic control | EGJ | NR | 40mL | Intraoperative | Immediately after myotomy |
| Yoo (2019) | Transoral, general anesthesia; under endoscopic control | EGJ | NR | 30-40mL | Preoperative | 1 month |
| Amundson (2023) | Transoral, general anesthesia; under endoscopic control | EGJ | 30-60s | 30-40mL | Intraoperative | Immediately after myotomy |
| DeWitt (2022) | Transoral, general anesthesia; under endoscopic control | EGJ | NR | NR | Preoperative | 6-12 months |
| Knight (2022) | Transoral, general anesthesia; under endoscopic control | EGJ | 30s | 40mL | Intraoperative | Immediately after myotomy |
| Teitelbaum (2014) | Transoral, general anesthesia; under endoscopic control | EGJ | NR | 30-40mL | Intraoperative | Immediately after myotomy |

YOP: Year of Publication; EGJ: Esophagogastric Junction; NR: Not Reported.
